# A genetic linkage map for the saltwater crocodile (*Crocodylus porosus*)

**DOI:** 10.1186/1471-2164-10-339

**Published:** 2009-07-29

**Authors:** Lee G Miles, Sally R Isberg, Travis C Glenn, Stacey L Lance, Pauline Dalzell, Peter C Thomson, Chris Moran

**Affiliations:** 1 Faculty of Veterinary Science, University of Sydney, NSW 2006, Australia; 2 Porosus Pty Ltd, PO Box 86, Palmerston, NT 0831, Australia; 3 Savannah River Ecology Laboratory, University of Georgia, PO Drawer E, Aiken, SC 29802, USA; 4 Department of Environmental Health Science, University of Georgia, Athens, GA 30602, USA; 5 South Eastern Area Laboratory Services, Randwick, NSW 2031, Australia

## Abstract

**Background:**

Genome elucidation is now in high gear for many organisms, and whilst genetic maps have been developed for a broad array of species, surprisingly, no such maps exist for a crocodilian, or indeed any other non-avian member of the Class Reptilia. Genetic linkage maps are essential tools for the mapping and dissection of complex quantitative trait loci (QTL), and in order to permit systematic genome scans for the identification of genes affecting economically important traits in farmed crocodilians, a comprehensive genetic linage map will be necessary.

**Results:**

A first-generation genetic linkage map for the saltwater crocodile (*Crocodylus porosus*) was constructed using 203 microsatellite markers amplified across a two-generation pedigree comprising ten full-sib families from a commercial population at Darwin Crocodile Farm, Northern Territory, Australia. Linkage analyses identified fourteen linkage groups comprising a total of 180 loci, with 23 loci remaining unlinked. Markers were ordered within linkage groups employing a heuristic approach using CRIMAP v3.0 software. The estimated female and male recombination map lengths were 1824.1 and 319.0 centimorgans (cM) respectively, revealing an uncommonly large disparity in recombination map lengths between sexes (ratio of 5.7:1).

**Conclusion:**

We have generated the first genetic linkage map for a crocodilian, or indeed any other non-avian reptile. The uncommonly large disparity in recombination map lengths confirms previous preliminary evidence of major differences in sex-specific recombination rates in a species that exhibits temperature-dependent sex determination (TSD). However, at this point the reason for this disparity in saltwater crocodiles remains unclear.

This map will be a valuable resource for crocodilian researchers, facilitating the systematic genome scans necessary for identifying genes affecting complex traits of economic importance in the crocodile industry. In addition, since many of the markers placed on this genetic map have been evaluated in up to 18 other extant species of crocodilian, this map will be of intrinsic value to comparative mapping efforts aimed at understanding genome content and organization among crocodilians, as well as the molecular evolution of reptilian and other amniote genomes. As researchers continue to work towards elucidation of the crocodilian genome, this first generation map lays the groundwork for more detailed mapping investigations, as well as providing a valuable scaffold for future genome sequence assembly.

## Background

Microsatellites are an excellent choice of genetic marker for genome mapping due to their hyper-variability and abundance throughout most vertebrate genomes [1]. Typing of microsatellite DNA loci by routine polymerase chain reaction (PCR) was developed almost 20 years ago [2-4], and has since facilitated the construction of dense genetic maps in many species. Genetic maps are valuable tools in numerous areas of genetic research, particularly for the localization and dissection of quantitative trait loci (QTL), and for comparative mapping between species. Comprehensive linkage maps have been developed using microsatellite markers in humans, many biomedical models, livestock, fish, birds, invertebrates, plants and other organisms [5-13]. Although genetic linkage maps have been developed for a broad array of species, no such maps exist for any crocodilians, or indeed any other non-avian member of the Class Reptilia.

Crocodilians are the sole surviving reptilian archosaur, a group of diapsids that include dinosaurs and other ancient reptiles that gave rise to birds [14]. Diverging from the evolutionary lineage that gave rise to mammals more than 300 million years ago [15-17], Reptilia represent a valuable intermediate evolutionary group placed between mammals and more distantly related vertebrate species such as fish [7,16]. Recent progress in the development of genomic resources for studies in reptiles has given impetus to comparative genomics aimed at understanding the evolution and structure of the reptilian genome [17-19]. Genome sequences are now available for two avian species [chicken [20], and zebra finch http://www.songbirdgenome.org/index.html], as well as for one non-avian reptile, the green anole (Order Squamata, http://www.broad.mit.edu/models/anole/). Work is also currently underway to sequence the genome of the painted turtle (Order Chelonia, http://www.genome.gov/10002154). Although the Order Crocodylia remains unrepresented, the generation of a comprehensive genetic map for a crocodilian will provide a significant step towards the elucidation of the crocodilian genome, providing a valuable scaffold for genome sequence assembly, and will be of intrinsic value to comparative mapping efforts aimed at understanding the molecular evolution of reptilian, as well as other amniote genomes.

From an economic perspective, crocodilians play an important role in modern agriculture, as well as forming a basis for tourism, with management programs in more than 40 nations worldwide [21]. The Australian crocodile industry produces farmed saltwater crocodiles (*Crocodylus porosus*) for the international skin trade. Although still an emerging livestock industry, the Australian crocodile industry, following the lead of other livestock industries, has recently developed a comprehensive genetic improvement program [22]. Research efforts have thus far focused on genetic and phenotypic parameter estimation for selection objectives and selection criteria required for multi-trait index selection [22-26]. However, this type of animal selection occurs with little or no knowledge of what is occurring at the DNA level. One of the major limitations to performance-based selection in crocodiles is the large generation interval, which is estimated to be 13 years [22]. To improve the rate of genetic gain currently achieved in the industry, particularly for traits that are difficult to measure such as disease resistance and sex limited traits, as well as other complex traits such as growth rate, animal survival and skin quality, trait-linked DNA markers will be necessary. Animal selection employing marker information will increase the rate of genetic gain by permitting early selection decisions to be made on large animal resources, thereby both increasing selection intensity and reducing the generation interval. The availability of a comprehensive linkage map, with markers evenly spaced across the genome, will facilitate the systematic searches necessary to identify genes affecting traits of economic importance, with the potential to incorporate marker information into the animal selection process using marker assisted selection (MAS) [27,28]. Accordingly, a genetic linkage map for the saltwater crocodile will have both scientific and commercial benefits.

Evidence of genetic linkage between ten microsatellites was previously reported for the saltwater crocodile by Isberg et al. [29] based on a limited number of genetic markers developed by Fitzsimmons et al. [30]. However, Miles et al. (2009a) [31] have since developed 253 novel polymorphic microsatellite markers for saltwater crocodiles, thus providing a sufficiently large marker resource for genome mapping. Besides the previous lack of suitable markers, another major reason why there are no genome maps for the Order Crocodylia is the difficulty in breeding informative predigrees from which DNA resources can be developed. Indeed, the lack of pedigreed animals has thus far thwarted efforts to generate a linkage map for other model reptiles, such as *Anolis*. Fortunately, the use of unitized breeding pens, coupled with detailed pedigree records in the Australian crocodile industry, has provided the pedigrees and complementary DNA resources necessary for the construction of the first crocodilian genetic map.

## Results

### Data integrity

Pedigree analyses identified incorrect parentage assignment for one of the six full-sib families comprising the *Linkage Reference Panel*. Unfortunately, the correct parents could not be identified within the population, and consequently this family was removed from the *Linkage Reference Panel *and future linkage analyses. The final *Linkage Reference Panel *consisted of five full-sib families comprising 83 individuals. Average heterozygosities for the parental animals are described in Miles et al. (2009a) [31].

### Linkage map characteristics

**Figure 1:**
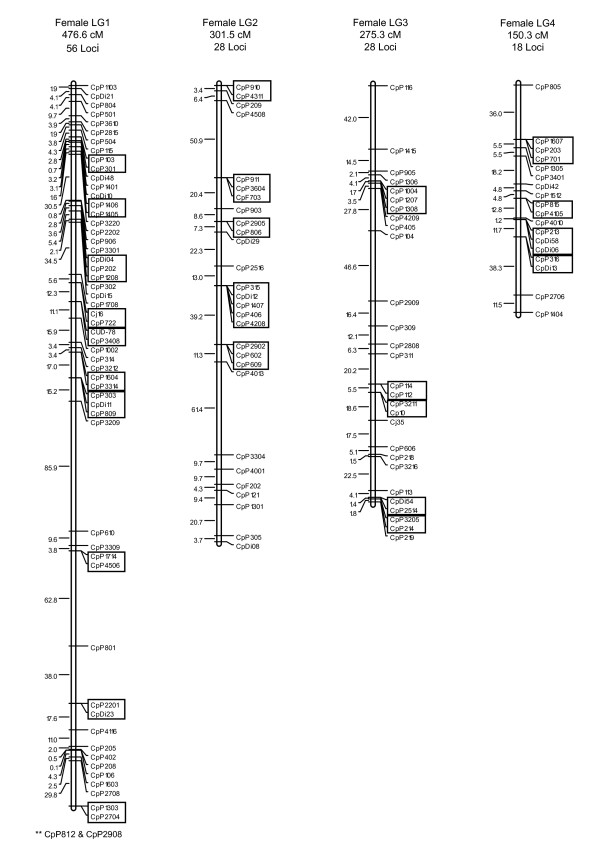
**Linkage groups 1 to 4 of the female-specific linkage map for *C. porosus***. The female-specific genetic map comprises 180 markers assigned to 14 linkage groups (LG1-LG14), and spans a total map length of 1824.1 cM. Linkage groups 1 to 4 are presented here. The number of loci and total estimated genetic lengths (cM) are provided above each respective linkage group. Locus nomenclature, map order, and inter-locus distances (Koasambi distances) are provided next to each of the linkage groups. Markers assigned to the same map location are boxed. Markers indicted by an asterisk ** could not be ordered within the existing map.

**Figure 2:**
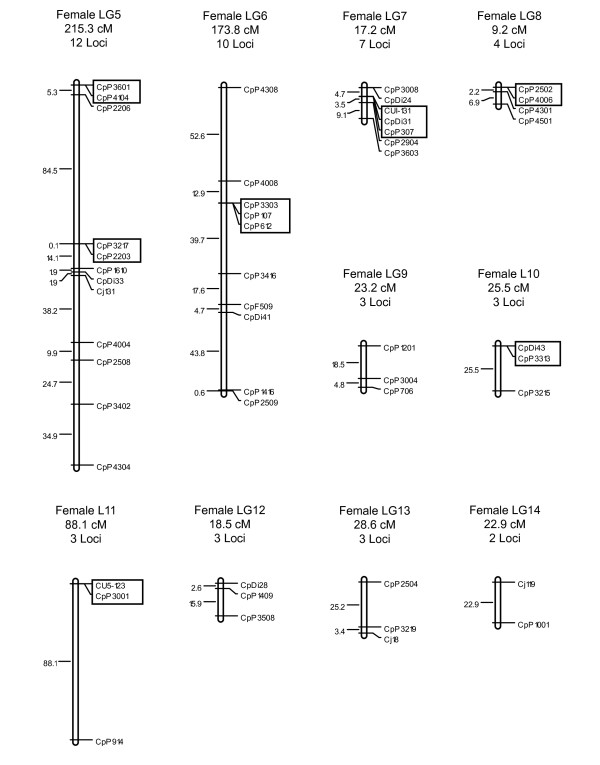
**Linkage groups 5 to 14 of the female-specific linkage map for *C. porosus***. The female-specific genetic map comprises 180 markers assigned to 14 linkage groups (LG1-LG14), and spans a total map length of 1824.1 cM. Linkage groups 5 to 14 are presented here. The number of loci and total estimated genetic lengths (cM) are provided above each respective linkage group. Locus nomenclature, map order, and inter-locus distances (Koasambi distances) are provided next to each of the linkage groups. Markers assigned to the same map location are boxed.

**Figure 3:**
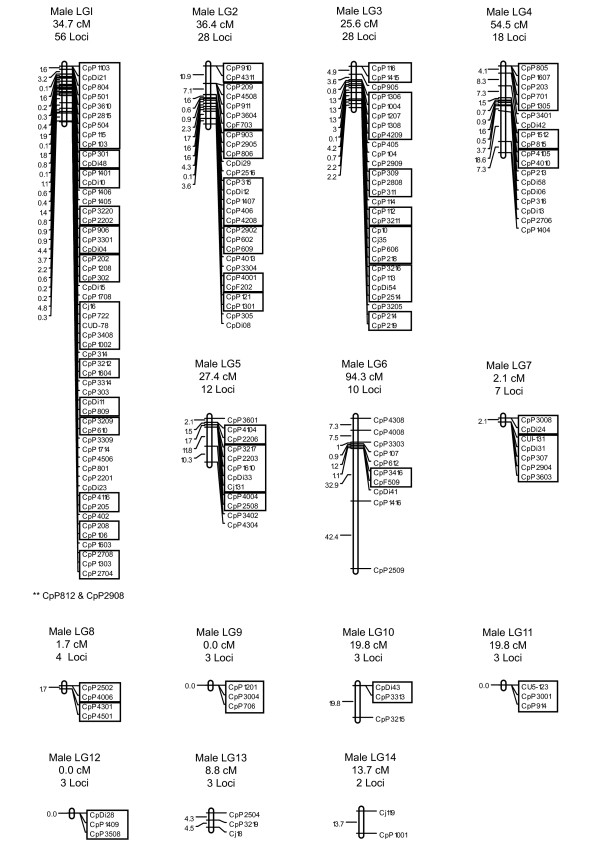
**The male-specific genetic linkage map for *C.porosus***. This genetic map comprises 180 markers assigned to 14 linkage groups (LG1-LG14), and spans a total map length of 319.0 cM. The number of loci and total estimated genetic lengths (cM) are provided above each respective linkage group. Locus nomenclature, map order, and inter-locus distances (Koasambi distances) are provided next to each of the linkage groups. Markers assigned to the same map location are boxed. Markers indicted by an asterisk ** could not be ordered within the existing map.

The average number of informative meioses for the 203 genotyped markers was 195, with a maximum of 848 and a minimum of 12. Two-point linkage analyses assigned 180 of the 203 typed microsatellites to 14 linkage groups at a LOD ≥ 3.0, with linkage group sizes ranging from two to 56 markers. Twenty-three microsatellites (CpDi16, CpF401, CpP1003, CpP1005, CpP1413, CpP1502, CpP1608, CpP1611, CpP201, CpP207, CpP2209, CpP2505, CpP2705, CpP2813, CpP2816, CpP3202, CpP3507, CpP4111, CpP607, CpP714, CpP715, CpP717, CpP1006) did not show significant linkage to any other markers. The total lengths for the female and male recombination maps were 1824.1 cM and 319.0 cM, respectively, indicating an uncommonly large disparity in sex-specific map lengths. Due to this large difference, no sex-averaged map is presented. In the female map, linkage group lengths varied from 9.2 to 476.6 cM, with inter-locus distances ranging from 0 to 88.1 cM, and an average distance of 14.9 cM. In the male map, linkage group lengths ranged from 0.0 to 94.3 cM, with inter-locus distances ranging from 0 to 42.4 cM, and an average distance of 4.0 cM. The current female and male maps contain 145 and 95 genetically separated positions, respectively. Further parameters for the saltwater crocodile linkage maps are summarized in Table 1. The spacing and ordering of markers along the 14 linkage groups for the female- and male-specific maps are presented in Figure 1, 2 and 3.

**Table 1:** Parameters of the *C. porosus *linkage map

									No. of intervals			
					
		Map Length (cM)			No. of positions		Average interval (cM)		15 – 30 cM		> 30 cM	
						
Linkage Group	No. of markers	Female	Male	F:M ratio	F	M	F	M	F	M	F	M
1	56*	476.6	34.7	13.7	42	28	11.3	1.2	4	0	5	0
2	28	301.6	36.4	8.3	18	13	16.8	2.8	3	0	3	0
3	28	275.3	25.6	10.8	22	13	12.5	2.0	6	0	2	0
4	18	150.3	54.5	2.8	12	12	12.5	4.5	1	1	2	0
5	12	215.3	27.4	7.9	11	6	19.6	4.6	1	0	3	0
6	10	171.8	94.3	1.8	8	9	21.5	10.5	1	0	3	2
7	7	17.2	2.1	8.2	4	2	4.3	1.1	0	0	0	0
8	4	9.2	1.7	5.4	3	2	3.1	0.9	0	0	0	0
9	3	23.2	0.0	8	3	1	7.7	0.0	1	0	0	0
10	3	25.5	19.8	1.3	2	2	12.8	9.9	1	1	0	0
11	3	88.1	0.0	8	2	1	44.1	0.0	0	0	1	0
12	3	18.5	0.0	8	3	1	6.2	0.0	1	0	0	0
13	3	28.5	8.8	3.3	3	3	9.5	2.9	1	0	0	0
14	2	22.9	13.7	1.7	2	2	11.5	6.9	1	0	0	0

total	180	1824.0	319.0	5.7	135	95	13.8	3.4	21	2	19	2

* Two markers could not be ordered within this linkage group

### Differences in sex-specific recombination maps

The exclusive mapping of co-dominant markers in the saltwater crocodile has permitted the direct comparison of inter-locus distances between sexes along the entire length of the recombination map. The female map length exceeds that of the male for every linkage group (Table 1), and the overall female map length is 5.7-fold greater than that of the male map. However, this disparity varies quite significantly among linkage groups. With the exception of those LGs where zero recombination is apparent in the male, the ratio of female-to-male map lengths ranges between 1.8:1 and 13.7:1 (Table 1), suggesting that although the high level of observed heterochiasmy exists across the entire map, it is not necessarily evenly distributed across the entire genome.

## Discussion

### An uncommonly large difference in sex-specific recombination rates

This study reports the first genetic map for a non-avian reptile. The most unusual feature of this map is the uncommonly large difference in female and male map lengths, with significantly lower recombination evident in the male. Evidence of heterochiasmy in Crocodylia was previously reported by Isberg et al [29], based on only three pair-wise linkages and a single linkage group comprising four loci exhibiting a female-to male recombination ratio of 2.8:1. The generation of a comprehensive linkage map incorporating 180 microsatellites presents far stronger support for this observation, with an estimated sex-specific recombination ratio exceeding 5.7:1 for females relative to males. The magnitude of this difference not only supports previous findings, but also establishes convincingly that it is a genome-wide phenomenon. Cytogenetic analysis of female and male meiosis will be required to further understand the basis of this uncommonly large difference.

### Recombination heterogeneity

Differences in recombination rates between sexes are not uncommon, and have been well documented in numerous vertebrate species. In mammals, this ratio has typically ranged between 1.0 and 2.0, with the heterogametic sex typically exhibiting the lower recombination rate [5,6,8,32,33], with several notable exceptions [10,34,35]. It has been suggested that birds show no sex-specific differences in recombination [36], whilst in other non-mammalian and non-avian vertebrates species, the sex-specific recombination ratio has been shown to exceed 2:1. Fish species are reported to exhibit the most extreme levels of heterochiasmy among vertebrates, with average ratios of 7.4:1 reported for the Japanese flounder [37], 8.26:1 reported for Atlantic salmon [38], and a ratio in excess of 10:1 reported for the Western Australian seahorse when four microsatellites were compared [39]. Although the average disparity for saltwater crocodiles did not exceed these previous reports, the disparity between female and male recombination maps lengths reached an upper limit as high as 13.7:1 for LG1. This evidence suggests that crocodilians exhibit one of the greatest disparities in recombination frequencies between sexes among vertebrate species. This finding also adds to the empirical evidence that fish and non-avian reptiles exhibit higher levels of heterochiasmy compared with mammals and birds. Moreover, the current saltwater crocodile map provides a further exception to the Haldane-Huxley rule [40,41], which purports that the reduced recombination frequency is characteristically observed in the heterogametic sex, since crocodilians exhibit temperature-dependent sex determination (TSD) and lack classical sex chromosomes. It would appear that heterochiasmy is in fact unrelated to the sex chromosomes as previous theories have implied, but presumably relates to other major differences in male and female meiosis.

Although the reason for the disparity in map distances between male and female saltwater crocodiles is thus far unexplained, several theories have been proposed for other vertebrate species. In eutherian mammals, it has been proposed that differences in the rate of male and female recombination might be related to the sex-specific environment in which the germ cell finds itself, as opposed to the genotype [42]. Hassold and Hunt [43] proposed that temporal differences in the initiation and progression of meiosis in males and females could affect recombination distribution and frequency between sexes. Alternatively, Tease and Hulten [44] proposed that differences in the pairing and synapsis of homologs at meiosis cause spermatocytes and oocytes to have different exchange patterns. Several studies using sex-reversed animals or meiocytes have investigated the relationship between phenotypic and genotypic sex on synaponemal complex (SC) length, chiasma distribution and recombination frequency, and all have reported the consistent association of sex-specific differences with phenotypic sex. In particular, Lynn et al. [42,45] showed that the rate and pattern of recombination in meiocytes from XY sex-reversed and XO female mice and humans were virtually identical to those in normal XX females. Similarly, sex-reversal studies in fish species have shown that phenotypic sex, rather than genotype, determines SC length, chiasma distribution, and recombination frequency, regardless of the sex determining system (i.e. XX/XY or WZ/WW [46,47]). SC length has been shown to be correlated with recombination frequency in placental mammals [45], whilst no clear relationship appears to exist between the sexual dimorphism of SC length and recombination frequency in fish species [46]. Similar cytogenetic studies should be undertaken in crocodilians, and other species with TSD, to determine whether sex-specific differences in recombination are attributable to differences in the architecture of SC between males and females.

### Map construction and genome coverage

The number of markers that showed significant linkage (LOD ≥ 3.0) with at least one other marker was high (88.7%). Of the 23 loci that remain unassigned, more than half had relatively low numbers of informative meioses and hence less power. Although fourteen linkage groups were identified, based on the karyotype available for the saltwater crocodile (2n = 34; [48]), at least three chromosomes still remain unrepresented. This information indicates that the true map length will be larger than that reported in the current map, therefore exceeding current estimates of 1824.1 and 319.0 cM for females and males, respectively. As additional markers are mapped to locations beyond the terminal markers of each linkage group, it will be of interest to note whether there is an inflation of the male recombination map length as a result of distal localization of chiasmata. Similar observations have been reported in the female fat-tailed dunnart, *Sminthopsis crassicaudata*, as a result of distal localization of chiasmata in all of the autosomes [49]. Certainly, clustering of chiasmata at the ends of chromosomes, coupled with interstitial chromosomal regions devoid of chiasmata in the male, would explain the significantly reduced size of the male recombination map compared with the female. However, with the data currently available, the cause of this disparity remains unclear.

While this framework map is reasonably comprehensive, it will undoubtedly evolve with the addition of more markers, allowing the establishment of linkage on unrepresented chromosomes, and refining interval orders for those existing linkage groups. Ideally, the addition of further microsatellites would be desirable due to their high levels of heterozygosity. However, microsatellites are expensive and labor intensive to generate. Moreover, reported low levels of repetitive sequence in non-avian reptiles may limit the rapid development of a saturated crocodilian genetic map with microsatellites alone [19]. Fortunately, recent advances in next generation sequencing technologies, coupled with the production of high-density microarray systems for highly multiplexed genotyping, have made genome-wide genotyping with thousands of single nucleotide polymorphism (SNP) markers possible [50], thus providing an efficient and cost-effective means of saturating genome maps. The generation and mapping of a saltwater crocodile SNP resource to refine the framework map is anticipated in the near future.

### Physically anchoring the *C. porosus *linkage map

The 14 linkage groups are not yet assigned to saltwater crocodile chromosomes. However, the recent generation of a Bacterial Artificial Chromosome (BAC) library for the saltwater crocodile (further information available at http://www.mgel.msstate.edu/dna_libs.htm) will facilitate the anchoring of the linkage map in the near future. The localization of BAC clones containing markers terminally located within linkage groups to saltwater crocodile chromosomes via fluorescent *in situ *hybridization (FISH) will not only permit the anchoring and orientation of linkage groups, but will also provide an indication of the level of genome coverage. This work is already underway.

### Linkage mapping and QTL analysis in the crocodile

In spite of future plans to refine the saltwater crocodile map, the current linkage map is sufficiently dense to facilitate preliminary systematic genome searches for QTL affecting economically important traits, as well as traits of evolutionary significance in farmed saltwater crocodiles. Marker information and phenotypic profiles obtained from QTL studies may, in the future, be incorporated into animal breeding programs through marker assisted selection (MAS). The ability to select replacement breeding stock based on marker genotypes at day of hatch would significantly improve the genetic improvement system by discounting the need to performance test animals prior to selection (particularly for sex-limited traits and those which are expressed late in life). This would also increase the intensity of selection and improve the overall rate of genetic gain by expediting selection information from day of hatch. This framework map is the first step towards the identification of QTL in farmed saltwater crocodiles. However, the accuracy of map interval order is of intrinsic importance to the dissection of complex quantitative traits [51]. Thus, continued map refinement will be vital to the future utility of the saltwater crocodile map for the development of MAS tools for the crocodile industry.

### Potential for comparative mapping of Crocodylia

Of the 180 microsatellites incorporated in this map, more than 70 have also been cross-amplified in as many as 18 non-source species of Crocodylia [52]. The high success of cross-amplification (more than 90% success for *Crocodylus *species) opens up the possibility for comparative mapping among the 14 extant Crocodilidae species, as well as between different genera of Crocodylia. Comparative mapping studies would permit researchers to draw upon the map information now available for the saltwater crocodile, and apply it to other crocodilians. For example, the genotypic determinant of a valuable phenotype in one species could be directly tested for its involvement in a similar phenotype in another species [33]. Furthermore, investigation of map synteny between species could provide valuable insights into genome organization and chromosome evolution in Crocodylia and other taxa [53]. Accordingly, this first-generation linkage map not only represents a valuable resource for researchers working with saltwater crocodiles, but also for crocodilian researchers and evolutionary biologists collectively.

## Conclusion

This linkage map for the saltwater crocodile genome represents the first genetic map for a crocodilian, or indeed any other non-avian reptile, and reveals major differences in genome-wide sex-specific recombination rates in a species that exhibits temperature-dependent sex determination (TSD). This framework map lays the groundwork for future detailed mapping studies of Crocodylia, in which additional microsatellites, SNPs and other markers will be added for rapid construction of next generation high-density maps. Given the high level of success of cross-species amplification reported for the mapped microsatellites [52], the existing map presents a valuable opportunity to conduct comparative mapping investigations aimed at understanding genome organization and patterns of genome evolution in Crocodylia. Furthermore, while it will be necessary to increase marker density before conducting detailed QTL mapping investigations, this first generation map is sufficiently dense to facilitate preliminary systematic genome searches to identify QTL and genomic regions affecting traits of economic importance in farmed crocodiles, as well as traits of ecological importance in wild populations. The *Porosus Mapping Resource *has been invaluable for the construction of the framework map, and it will hopefully continue to support map refinement in the future. DNA from this resource will be made available to research groups seeking to place additional public domain markers on the existing framework for map refinement.

## Methods

### Mapping population

The long generation interval (13 years) for saltwater crocodiles [22], coupled with the relative infancy of the industry, means that no deep pedigrees are available for making DNA resources for mapping. The mapping panel developed for this study, the *Porosus Mapping Panel*, was therefore derived from a simple two-generation pedigree. Parents chosen for inclusion in this resource were long-term, known-breeding pairs housed in unitized pens at the Darwin Crocodile Farm, Northern Territory, Australia. The parents were wild-caught and assumed to be unrelated. Eggs from breeding pairs were collected and artificially incubated until hatch. Upon hatch, the offspring were uniquely marked using scute cuts [22]. Clutches arising from the 2005, 2006 and 2007 nesting seasons were included in this study. Capture, handling and blood sampling of crocodiles was approved by Australian Animal Ethic Committee, permit No. N00/8-2005/3/4177.

As the purpose of constructing a genetic linkage map is to facilitate whole genome scans for QTL, data from animals subsequently typed for QTL studies were integrated into the final linkage analysis. Consequently, the *Porosus Mapping Panel *can be divided into two sub-panels: the *Linkage Reference Panel *and the *QTL Resource Panel*.

#### Linkage Reference Panel

The *Linkage Reference Panel *consisted of 96 individuals from six full-sib families. This panel size was chosen to correspond with the 96-well plate platform used for both PCR and genotype analyses in order to streamline data generation and collection. All family cohorts in this panel were selected from 2007 offspring, and family sizes ranged from 14 to 18 individuals. This resource was typed for all available polymorphic loci to construct the framework linkage map.

#### QTL Resource Panel

The *QTL Resource Panel *consisted of 482 individuals from ten full-sib families comprising offspring from the 2005, 2006 and 2007 cohorts. Individual families included in the QTL resource were selected based on the large variation in residuals for traits of interest, after accounting for fixed effects, using the restricted maximum likelihood (REML) models described in Isberg et al. [24-26]. Family sizes ranged from 13 to 89 individuals. Eighty-three of the individuals from this panel were also present in the *Linkage Mapping Panel*. The 399 individuals that did not appear in the *Linkage Mapping Panel *were typed for 71 microsatellite loci selected for their even distribution across the resulting framework linkage map based on the *Linkage Reference Panel *alone.

### DNA extraction

All blood samples were collected with EDTA (ethylenediaminetetraacetic acid) syringes from the cervical sinus using the method described in Lloyd and Morris [54], and immediately frozen for later processing. DNA was isolated and purified from *C. porosus *blood using a phenol-chloroform extraction protocol adapted from Sambrook et al. [55].

### Microsatellite genotyping

In total, 262 polymorphic microsatellites [30,31] were genotyped on the *Linkage Reference Panel*. PCR reactions for each marker were performed in 12.5 μl-volumes using PTC-100 (MJ Research) and GeneAmp PCR System 9700 (Applied Biosystems) thermocyclers. Final concentrations for optimized reactions were 10 mM Tris pH 8.4, 50 mM KCl, 0.5 μM unlabeled primer, 0.05 μM tag labeled primer, 0.45 μM universal dye labeled primer, 2.0 mM MgCl_2_, 0.5 mM dNTPs, 0.5 U units JumpStart Taq DNA Polymerase (Sigma), and approximately 20 ng DNA. Universal CAG-primers were labeled with either VIC, 6-FAM or NED fluorescent dyes, as previously described in Miles et al. (2009a) [31]. Reactions were placed on one of two stratified touchdown profiles [56], with each profile encompassing a 10°C span of annealing temperatures (ranges: 65-55°C and 55-45°C), according to the optimal conditions identified for each respective primer [31]. PCR amplicons for each of the respective fluorochromes were pooled (VIC, 6-Fam and NED) and analyzed on an ABI 3130xl or 3730xl automated DNA sequencer. Raw data were imported into Genemapper version 4.0 (Applied Biosystems) for genotype analysis. Genotypes for each microsatellite locus were scored, and exported into a single data set via a custom BioPython script.

Errors in genotype data are known to considerably inflate genetic map distances [57]. To circumvent genotype errors in the current dataset, all genotypes were verified by an independent third party. The PREPARE function in CRIMAP was also used to flag spurious genotypes that departed from the expected Mendelian segregation patterns. Any discordant genotypes were either retyped or removed from the dataset. Of the 262 markers genotyped, 203 performed satisfactorily enough to be included in subsequent linkage analyses.

### Dataset integrity

Prior to linkage analyses, genotype data were analyzed using the software Cervus version 3.0 [58] to ensure pedigree integrity and correct parentage [59]. Assuming a typing error rate of 0.01, and strict confidence levels of 95%, probabilities of exclusion were estimated for all individuals in the mapping population.

### Linkage map construction

CRI-MAP v3.0 [60] was used to construct the saltwater crocodile linkage map. Markers were assigned to linkage groups based on linkage supported by two-point LOD scores ≥ 3.0, and later ordered within these groups using multi-point linkage analyses employing a heuristic approach. LOD ≥ 3.0 was chosen as the minimum statistical support criterion for ascertaining locus order of framework loci within linkage groups using the BUILD option. This threshold was subsequently reduced to LOD ≥ 2.0 to place the remaining loci within the framework map. For markers not supported with a LOD ≥ 2.0, probable map locations were determined using the ALL function. Upon incorporation of all possible markers, the FLIPS option was used to ascertain the best possible locus order by looking at all possible permutations for up to six adjacent loci within the resulting map. Once the most likely map order had been derived, sex-specific map distances in centimorgans (cM) were estimated for each linkage group using the Kosambi [61] mapping function. Maps were drawn using the software MapDraw version 2.2 [62]. Linkage group nomenclature was assigned in order of descending number of markers per linkage group.

## Competing interests

The authors declare that they have no competing interests.

## Authors' contributions

LGM carried out the marker development, contributed to the pedigree and DNA panel development, generated the genotype data, performed the linkage analyses and drafted the manuscript. SRI conceived the study, participated in its design and coordination, and was the primary impetus behind the pedigree development. TCG participated in the marker development, genotype data generation, and helped to draft the manuscript. SLL helped perform pedigree integrity analysis and contributed to the genotype data generation. PD participated in the experimental design and mapping analysis. PCT participated in the experimental design and provided statistical support. CM participated in the study's conception, design and coordination, and helped to draft the manuscript. All authors read and approved the final manuscript.
